# Persulfidation of Human Cystathionine *γ*-Lyase Inhibits Its Activity: A Negative Feedback Regulation Mechanism for H_2_S Production

**DOI:** 10.3390/antiox13111402

**Published:** 2024-11-15

**Authors:** Guanya Jia, Heng Li, Haisheng Gan, Jun Wang, Zhilong Zhu, Yanxiong Wang, Yongyi Ye, Xiaoya Shang, Weining Niu

**Affiliations:** School of Life Sciences, Northwestern Polytechnical University, Xi’an 710072, China; jiagy@mail.nwpu.edu.cn (G.J.); aheng@mail.nwpu.edu.cn (H.L.); hsgan@mail.nwpu.edu.cn (H.G.); junw@mail.nwpu.edu.cn (J.W.); zhilongzhu_2019@mail.nwpu.edu.cn (Z.Z.); wangyanxiong2023@mail.nwpu.edu.cn (Y.W.); yyye@mail.nwpu.edu.cn (Y.Y.); loyamuyu@nwpu.edu.cn (X.S.)

**Keywords:** cystathionine γ-lyase, hydrogen sulfide, persulfidation, post-translational modification, negative feedback regulation

## Abstract

Cystathionine γ-lyase (CSE) is the second enzyme in the trans-sulfuration pathway that converts cystathionine to cysteine. It is also one of three major enzymes responsible for the biosynthesis of hydrogen sulfide (H_2_S). CSE is believed to be the major source of endogenous H_2_S in the cardiovascular system, and the CSE/H_2_S system plays a crucial role in a variety of physiological and pathological processes. However, the regulatory mechanism of the CSE/H_2_S system is less well understood, especially at the post-translational level. Here, we demonstrated that the persulfidation of CSE inhibits its activity by ~2-fold in vitro. The loss of this post-translational modification in the presence of dithiothreitol (DTT) results in a reversal of basal activity. Cys137 was identified as the site for persulfidation by combining mass spectrometry, mutagenesis, activity analysis and streptavidin–biotin pull-down assays. To test the physiological relevance of the persulfidation regulation of CSE, human aortic vascular smooth muscle cells (HA-VSMCs) were incubated with vascular endothelial growth factor (VEGF), which is known to enhance endogenous H_2_S levels. Under these conditions, consistent with the change tendency of the cellular H_2_S level, the CSE persulfidation levels increased transiently and then gradually decreased to the basal level. Collectively, our study revealed a negative feedback regulation mechanism of the CSE/H_2_S system via the persulfidation of CSE and demonstrated the potential for maintaining cellular H_2_S homeostasis under oxidative stress conditions, particularly in tissues where CSE is a major source of H_2_S.

## 1. Introduction

Hydrogen sulfide (H_2_S) is one of three gasotransmitters (along with NO and CO) that modulate a variety of biological processes in the cardiovascular system, central nervous system and gastrointestinal tract [1,2,3,4]. In mammals, the biogenesis of H_2_S involves the trans-sulfuration pathway enzymes cystathionine *γ*-lyase (CSE) and cystathionine *β*-synthase (CBS). A third enzyme, 3-mercaptopyruvate sulfur transferase (3-MST), can also contribute to H_2_S biogenesis using 3-mercaptopyruvate as a substrate in the presence of reductants. 3-MST is located in the mitochondria and cytosol, whereas CSE and CBS are predominantly cytosolic [5,6]. In addition, the distribution of these three enzymes is tissue-specific, and CSE is the dominant H_2_S-generating enzyme in the cardiovascular system, specifically in smooth muscle cells, vascular endothelial cells and cardiomyocytes [7]. Considering the important roles of the CSE/H_2_S system in regulating cellular functions, a deeper understanding of the regulatory mechanism of the CSE/H_2_S system will be beneficial to the development of novel strategies to target CSE and regulate H_2_S biogenesis.

Human CSE is the second enzyme in the trans-sulfuration pathway and catalyzes the cystathionine to produce cysteine, which is the substrate in the synthesis of glutathione (GSH). Additionally, CSE can also catalyze various other reactions to generate H_2_S using cysteine or homocysteine as a substrate [8,9]. However, at physiologically relevant concentrations, cysteine is predicted to serve as the major H_2_S-generating substrate for CSE [10,11]. Maintaining the steady-state level of H_2_S requires tight control between its biosynthesis and catabolism. Therefore, the activities of H_2_S-producing enzymes should be finely regulated under different physiological conditions. Excess H_2_S in cells is mainly eliminated by a dedicated mitochondrial sulfide oxidation pathway, which includes sulfide quinone reductase (SQR), persulfide dioxygenase (ETHE1), rhodanese and sulfite oxidase [5,12].

The regulation of CSE is not well understood in comparison to CBS, another H_2_S-generating enzyme. In fact, studies on CSE regulation are very few and mainly focus on the regulation mechanisms at the transcriptional level [13,14,15,16,17,18]. The regulatory mechanisms of CSE activity at the protein level have not yet been adequately explored. Human CSE is a homotetramer with each subunit containing two domains: a larger pyridoxal 5′-phosphate (PLP) binding domain for catalysis and a smaller C-terminal domain. Each subunit contains ten cysteine residues, including two conserved CXXC motifs, Cys252-X-X-Cys255 and Cys307-X-X-Cys310 [9,19]. Our previous research indicated that CSE activity can be regulated by a reversible redox change in the Cys252-X-X-Cys255 motif involving a disulfide–dithiol reaction under different cellular redox conditions [20]. Recently, Liu et al. reported that O-GlcNAcylation of CSE at amino acid residue Ser138 promotes enzymatic activity to produce H_2_S, and the increased level of H_2_S represses trophoblast syncytialization by hampering androgen receptor (AR) dimerization [21].

S-nitrosation and persulfidation of CSE have also been reported. Luo et al. demonstrated that homocysteine accumulation in mice augments the nitration level of CSE, thus inhibiting its H_2_S-producing activity, although the nitrated amino acid residues remain unidentified [22]. In another study, the amino acid residue Cys137 was identified as contributing to S-Nitrosoglutathione (GSNO)-mediated CSE inhibition [23]. Additionally, a previous study indicated that CSE from mouse liver lysate can be persulfidated under physiological conditions [24]. Most recently, Araki et al. reported that the polysulfidation modification of rat CSE at the Cys136 residue (137 in humans) inhibits the cysteine persulfide (Cys-SSH)-producing activity of the enzyme during cystine metabolism [25]. In human CSE, five cysteine residues (Cys84, Cys109, Cys229, Cys252 and Cys307) have been identified as the predominant targets for persulfidation, and this cysteine modification appears to affect the H_2_S-producing activity of CSE [22]. However, critical cysteine residues for CSE persulfidation modification reported in the literature are inconsistent. The molecular mechanism and physiological relevance of CSE persulfidation have not been fully explored.

Persulfidation is a new post-translational modification similar to nitrosation. Persulfidation occurs in reactive cysteine residues of the targeted proteins and results in the conversion of a thiol group (-SH) of cysteine to an -SSH group [12,26]. Increased levels of cellular H_2_S production could induce persulfidation on receptors or enzymes, which are involved in a wide range of metabolic pathways and cellular functions [24,27,28]. Our previous study demonstrated that H_2_O_2_-mediated oxidative stress stimulates H_2_S production via the enhancement of CSE activity in human aortic vascular smooth muscle cells (HA-VSMCs) incubated with exogenous vascular endothelial growth factor (VEGF) [20]. Furthermore, we found that the cellular H_2_S level peaked at 30 min after VEGF treatment and then gradually decreased to the basal level after 2 h, whereas no significant difference was found in the protein expression of CSE, suggesting that CSE could be post-translationally modified, leading to CSE inhibition. Product feedback inhibition is a common mechanism of enzyme activity regulation. For example, it has been observed that the activity of nitric oxide synthase is feedback-inhibited by its own product, nitric oxide (NO) [29]. Based on the experimental observation and analysis mentioned above, we speculated that increased levels of H_2_S in cells under oxidative stress conditions could feedback inhibit the H_2_S-producing activity of CSE, thus leading to a decrease in H_2_S levels. In the present study, by investigating the effect of persulfidation on the activity of CSE, we showcase a novel negative feedback regulatory mechanism of the CSE/H_2_S pathway, and this feedback regulation may contribute to the maintenance of H_2_S homeostasis in cells.

## 2. Materials and Methods

### 2.1. Materials

The anti-cystathionine γ-lyase (CSE) antibody and the anti-persulfide dioxygenase (ETHE1) antibody were purchased from ABclonal (Wuhan, China). The fluorescent probe SF7-AM was obtained from Cayman (Ann Arbor, MI, USA). The fluorescent dye 2′,7′-dichlorodihydrofluorescein diacetate (DCFH-DA) for reactive oxygen species (ROS) detection was obtained from Solarbio (Beijing, China). The fluorescent dye 3-amino, 4-aminomethyl-2′,7′-difluorescein, diacetate (DAF-FM DA) for nitric oxide (NO) detection was purchased from Beyotime Biotechnology (Shanghai, China). The fluorescent dye 3′,6′-di(*O*-thiosalicyl)fluorescein (SSP4) for determination of inorganic polysulfide species in H_2_S solutions was obtained from Dojindo Laboratories (Kumamoto, Japan). Fetal bovine serum was obtained from Biological Industries (Kibbutz Beit Haemek, Israel). EZ-Link Maleimide-PEG2-Biotin (MPB) and streptavidin agarose resin were obtained from Thermo Fisher Scientific (Waltham, MA, USA). The cell counting kit-8 (CCK-8) was purchased from Dojindo (Kumamoto, Japan).

### 2.2. Recombinant Protein Expression

The mutants C109S, C137S, C172S, C252S and C255S were constructed by site-directed mutagenesis recombinant human CSE protein and were expressed as previously described, with a few modifications [20,30]. *E. coli* BL21(DE3) cells harboring the expression vector for His-tagged CSE were cultured in LB medium until the optical density at 600 nm reached 0.6–0.8. Protein expression was induced by the addition of 0.1 mM isopropyl β-d-1-thiogalactopyranoside (IPTG), and cells were cultured for 12 h at 25 °C. Cell pellets were resuspended in buffer A (50 mM PBS buffer, pH 7.4, 500 mM NaCl, 500 mg/L lysozyme) supplemented with a protease inhibitor tablet (Roche, Basel, Switzerland). After incubation on ice for 1 h, the resuspended cells were sonicated with an Ultrasonic Cell Disruption System (Scientz, Ningbo, China). The supernatant was collected by centrifugation at 12,000 rpm for 15 min at 4 °C and then loaded onto a HisTrap FF column (GE Healthcare, Fairfield, CT, USA) that had been pre-equilibrated with buffer B (50 mM PBS buffer, pH 7.4, 500 mM NaCl, 30 mM imidazole, 20 µM PLP). The elution was performed with 300 mM imidazole in 50 mM PBS buffer at pH 7.4. The fractions containing CSE were desalted using a HiPrep desalting column (GE Healthcare, USA) that had been pre-equilibrated with 50 mM PBS buffer at pH 7.4. Fractions containing the purified recombinant CSE were pooled, concentrated by a 30 kDa ultrafiltration tube (Merck Millipore, Billerica, MA, USA) and stored at −80 °C.

### 2.3. CSE Activity Assay

The H_2_S-producing activity of CSE using cysteine as a substrate was determined as previously described [11,31,32]. The reaction process of H_2_S with lead nitrate to generate lead sulfide was monitored at 390 nm in a multifunctional microplate reader. An extinction coefficient of 5500 M^−1^ cm^−1^ at 390 nm was used to calculate the lead sulfide concentration [31]. Briefly, the enzyme reaction mixtures (200 µL per reaction) containing 50 mM HEPES buffer at pH 7.4, 20 mM L-cysteine, 0.4 mM lead nitrate and 20 µM PLP were preincubated at 37 °C for 5 min in a 96-well plate; then, the reaction was started by the addition of 10 µg of CSE protein and monitored at 37 °C for 10 min.

The modification of wild-type CSE and variants was performed as follows. To specifically oxidize the redox-active thiols, recombinant CSE (1 mg/mL) was incubated with 1 mM diamide (Sigma-Aldrich, St. Louis, MO USA) for 30 min at room temperature, and then the reaction mixture was desalted to remove the diamide using a desalting column. Subsequently, the persulfidation of CSE was prepared by incubating the diamide-treated CSE (1 mg/mL) with 200 µM Na_2_S for 30 min at room temperature. For persulfidation with H_2_O_2_, purified CSE protein (1 mg/mL) was treated with 20–200 µM H_2_O_2_ for 30 min at room temperature, and then 100–1000 µM Na_2_S (five equivalents relative to H_2_O_2_) was added to the reaction mixture for 30 min. The persulfidated CSE was incubated with 10 mM DTT for 30 min to reduce the modified cysteine residues. All the modified samples were desalted to remove diamide, Na_2_S or DTT. After complete removal of DTT, the active vicinal thiols Cys252 and Cys255 in CSE were reoxidized to form disulfide by bubbling air through the solution (supplemented with 50 µM CuSO_4_) for 1 h [20,33,34,35]. The protein concentration was measured using a BCA protein assay kit (TransGen Biotech, Beijing, China).

### 2.4. Mass Spectrometry Analysis

Recombinant CSE (1 mg/mL) was treated with 1 mM diamide for 30 min. After diamide removal via a desalting column, 200 µM Na_2_S was added to the protein solutions, followed by incubation for 30 min at room temperature. Next, the sulfhydryl group (-SH) and persulfidation group (-SSH) of the samples were alkylated with 25 mM iodoacetamide in the dark for 1 h. The reaction solution was ultrafiltered and buffer-exchanged with 50 mM ammonium bicarbonate. The samples were digested using trypsin or chymotrypsin at 37 °C for 16 h, and the digested peptides were acidified with 10% formic acid and used for LC/MS-MS analysis. All analyses were conducted using a Thermo Scientific Ultimate 3000 RSLCnano system with a Reprosil-Pur C18-AQ column (Dr. Maisch GmbH, Ammerbuch, Germany). The mobile phases were composed of 0.1% formic acid in water (A) and 0.1% formic acid in acetonitrile (B). The peptides were eluted using the following gradient: 5% B for 5 min; 5–50% B over 20 min; 50–90% B over 5 min; 90% B for 5 min; and 90–5% B over 10 min. The injection volume was 10 μL, and the flow rate was 0.3 μL/min. The peptides were detected using a Q Exactive hybrid quadrupole-Orbitrap mass spectrometer (Thermo Finnigan, Somerset, NJ, USA) in positive electrospray ionization mode. Survey full scan MS spectra (from *m*/*z* 300–1800) were acquired with a resolution of 70,000. The top 20 signal intensity ions were fragmented using collision-induced dissociation (CID). Mass spectrometric data were searched against the National Center for Biotechnology and Information database. Search parameters included a precursor ion mass tolerance of 20 ppm, fragment ion mass tolerance of ±0.02 Da and variable amino acid modifications: protein N-terminal acetylation, oxidation of methionine and carbamidomethylation of cysteines.

To determine cysteine residues that undergo oxidation to form cysteine sulfenic acid (Cys-SOH) by H_2_O_2_, the wild-type CSE protein (1 mg/mL) was incubated with 50 µM H_2_O_2_ for 30 min at room temperature. Subsequently, dimedone (5 mM) was added to the sample for sulfenic acid labeling. Cysteine sulfenic acid modification was detected by mass spectrometry analysis as described above.

### 2.5. Images of Endogenous H_2_S, NO and ROS in Live Cells

The endogenous H_2_S in HA-VSMCs was detected using the hydrogen sulfide fluorescent probe SF7-AM [36]. HA-VSMCs were cultured in DMEM with 10% fetal bovine serum at 37 °C in a humidified atmosphere of 5% CO_2_. When 80% confluence was reached in 6-well plates, VEGF was added to a final concentration of 50 ng/mL, followed by incubation for 15, 30, 60 and 120 min. Subsequently, the cells were washed three times with prewarmed PBS, after which fresh medium containing 2.5 μM SF7-AM was added. After 30 min of incubation, the cells were washed with warmed PBS and imaged by an inverted fluorescence microscope (Leica, Nussloch, Germany). The endogenous NO was detected using the fluorescent probe DAF-FM DA (5 µM). The endogenous ROS was detected using the fluorescent dye DCFH-DA (10 µM). Images of living cells were acquired in six different fields. ImageJ was employed to analyze the images.

### 2.6. Determination of Free Thiols in CSE

The CSE was precipitated with 10% (*v*/*v*) trichloroacetic acid (TCA) at 4 °C for 30 min. The precipitate was collected by centrifugation at 12,000 rpm for 10 min at 4 °C. Subsequently, the precipitated protein was washed with ice-cold acetone to remove traces of TCA and redissolved in 100 mM phosphate buffer containing 3% (*w*/*v*) SDS, pH 7.0. To determine the number of free thiol groups in CSE, the unmodified and modified CSE protein (0.5 mg/mL) was titrated with 1 mM 5,5′-dithiobis(2-nitrobenzoic acid) (DTNB). Control samples contained 1 mM DTNB in the same buffer but were devoid of CSE protein. The mixtures were incubated for 10 min at room temperature, and the samples were analyzed at 412 nm by a multifunctional microplate reader using an extinction coefficient of 13,600 M^−1^cm^−1^ [31,37,38]. The protein concentration was determined using a BCA assay kit (TransGen Biotech, China).

### 2.7. Detection of Protein Persulfidation

The persulfidation levels of the wild-type and variant forms of CSE were detected according to previous published work with a few modifications [39]. Recombinant CSE protein (1 mg/mL) was treated with 1 mM diamide for 30 min at room temperature. After removal of diamide by using a desalting column, 200 µM Na_2_S was added to the protein solutions, followed by incubation for 30 min at room temperature. Each sample was added to 20 mM iodoacetamide (IAM), followed by frequent shaking at room temperature for 1 h. Subsequently, the IAM was removed using a desalting column that had been pre-equilibrated with 50 mM HEPES buffer (pH 7.4). The IAM-treated CSE protein was incubated with 5 mM tris(2-carboxyethyl)phosphine (TCEP) at room temperature for 30 min, followed by the complete removal of TCEP using a desalting column. After the complete removal of TCEP, the active vicinal thiols Cys252 and Cys255 in CSE were reoxidized to form disulfide by bubbling air through the solution (supplemented with 50 µM CuSO_4_) for 1 h [20,33,34,35]. Subsequently, each sample was treated with 20 mM EZ-Link Maleimide-PEG2-Biotin (MPB) for 3 h at room temperature. The Maleimide-PEG2-Biotin was removed by the addition of cold acetone, and then the proteins were resuspended in 50 mM HEPES buffer (pH 7.4). The protein concentration was measured using a BCA protein assay kit, and each sample was subsequently diluted to reach a final protein concentration of 0.5 mg/mL. The biotinylated proteins were purified using streptavidin–agarose resin (50 µL resin/sample), which was then washed three times with TBST buffer (20 mM Tris–HCl, pH 7.4, 150 mM NaCl, 0.05% Tween 20) to eliminate nonspecific binding. The beads were finally boiled for 5 min in SDS-PAGE sample loading to elute all bound material for Western blot analyses.

To determine protein persulfidation in HA-VSMCs, cells were treated with VEGF (50 ng/mL) for 30, 60 and 120 min. Next, the cells were lysed by cyclic liquid nitrogen freezing–thawing in lysis buffer (50 mM HEPES, pH 7.4, 1% NP-40, 50 mM NaCl, 1 mM EDTA) supplemented with protease inhibitor cocktail (Roche). The supernatant was collected by centrifugation at 12,000 rpm for 10 min at 4 °C, and then the protein was alkylated with 20 mM IAM at room temperature for 1 h. Subsequently, the IAM was removed by the addition of cold acetone, and then the proteins were resuspended in 50 mM HEPES buffer (pH 7.4). After this step, the same protocol was applied as with the purified CSE protein samples. The persulfidated proteins were purified using streptavidin–agarose beads and analyzed by Western blot with CSE antibody. ImageJ was used to analyze the intensity of Western blot bands, and glyceraldehyde-3-phosphate dehydrogenase (GAPDH) served as a loading control.

### 2.8. Statistical Analysis

Data are represented as the mean ± SD. Unpaired Student’s t-test and one-way analysis of variance (ANOVA) were employed to determine the statistical significance using Graph Pad Prism 7.0. *p* < 0.05 was considered statistically significant.

## 3. Results

### 3.1. H_2_S Inhibits CSE Activity

**Figure 1:**
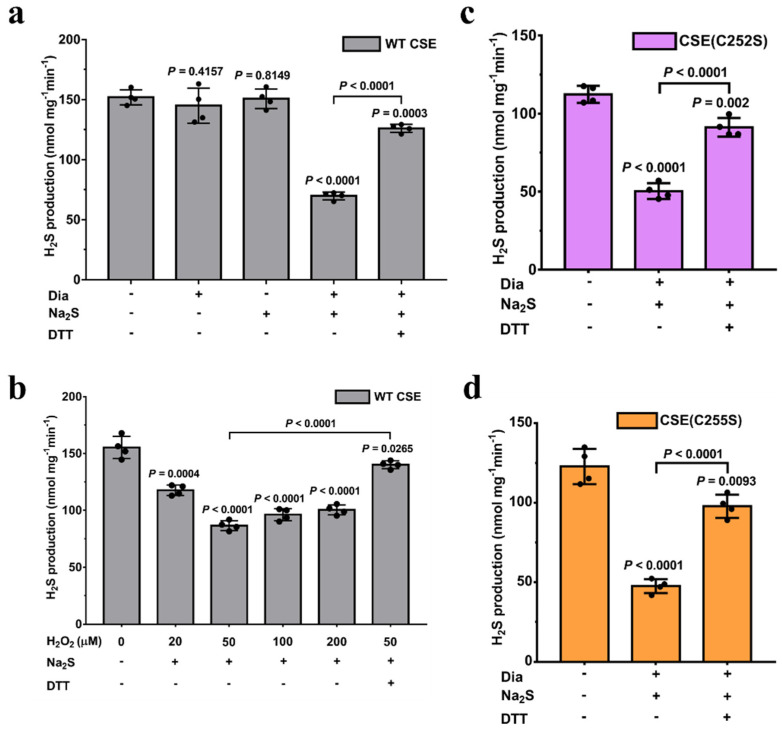
Effect of hydrogen sulfide on CSE activity. (**a**,**b**) Recombinant wild-type CSE (WT CSE) was incubated with diamide (1 mM) or H_2_O_2_ (20–200 µM) for 30 min, followed by the addition of Na_2_S for another 30 min of incubation. The modified CSE was incubated with 10 mM DTT for 30 min to reduce the modified cysteine residues. After complete removal of DTT, the active vicinal thiols Cys252 and Cys255 in the CXXC motif were reoxidized to form disulfide by bubbling air through the solution. CSE activity was measured using 20 mM *L*-cysteine as a substrate. The data points and errors are the means ± SDs (n = 4). Dia: diamide. (**c**,**d**) Recombinant CSE variants CSE (C252S) and CSE (C255S) were treated, and the activities were determined.

To test whether CSE activity can be affected by Na_2_S (an H_2_S donor), the purified CSE protein (1 mg/mL) was oxidized with the thiol-specific oxidant diamide (1 mM) to form mixed disulfides. Following diamide removal, the protein was treated with Na_2_S (200 µM) to persulfidate one or more susceptible thiol groups. The H_2_S-generating activity of wild-type CSE was measured using *L*-cysteine as a substrate. Persulfidation decreased the activity of wild CSE to 46% compared with untreated control samples (151.9 ± 3.12 nmol mg^−1^ min^−1^). The addition of DTT (10 mM) reversed the activity of persulfidated CSE to 83% of the control value (Figure 1a). Additionally, the CSE activity was not affected by the treatment with diamide or Na_2_S individually under these conditions. As the activity of CSE depends on the cofactor PLP, the effect of persulfidation on the PLP content in CSE was determined using ultraviolet–visible spectrophotometry. In the purified CSE, a specific absorption peak with *λ*max at ≈428 nm from PLP can be observed, and the characteristic absorption band was maintained on the persulfidated CSE, indicating no effect of persulfidation on the content of the PLP cofactor (Figure S1). Additionally, previous studies have shown that polysulfide contaminants formed in solutions of H_2_S could cause the persulfidation of proteins [12]. We measured the amount of inorganic polysulfide species in Na_2_S solutions (100–500 µM) using the fluorescent probe SSP4. No detectable levels of polysulfides were observed under the experimental conditions (Figure S2).

We next determined whether the treatment with H_2_O_2_ (a physiologically relevant oxidant) and H_2_S inhibits CSE activity through persulfidation modification. The activity of CSE decreased when treated with different concentrations of H_2_O_2_ (20–200 µM) for 30 min and exposed to Na_2_S, reaching the lowest at 50 µM H_2_O_2_, where the activity was 56% of the control value (Figure 1b). Beyond this concentration of H_2_O_2_, the activity of CSE slightly increased. Unlike treatment with diamide, a thiol-specific oxidant, which only results in the formation of disulfide bonds [40], treatment with H_2_O_2_ can oxidize the active thiols to generate sulfenic, sulfinic and sulfonic acids. Considering that the sulfinic and sulfonic acid cannot be modified by Na_2_S, the proportion of persulfidated CSE is expected to be lower in H_2_O_2_ versus diamide-treated samples. Actually, the activity of CSE treated with diamide and Na_2_S was lower than that treated with H_2_O_2_ and Na_2_S (Figure 1a,b). Hence, in subsequent experiments, diamide and Na_2_S were employed to modify the CSE. In addition, the activity of persulfidated CSE can be restored to 90% of the basal CSE activity in the presence of 10 mM DTT (Figure 1b).

Our previous study indicated that the active vicinal thiols, Cys252 and Cys255, in the CXXC motif can be oxidized to form disulfide by exposure to air oxygen in the absence of a reducing agent [20]. After the complete removal of DTT, the free thiols in the C252XXC255 motif are reoxidized to form disulfide by bubbling air through the solution in the presence of trace amounts of metal ions. The number of thiols in CSE protein was determined using a DTNB assay. A total of 10.1 ± 0.38 cysteines was modified in the reduced CSE, and the number decreased to 7.9 ± 0.26 in the reoxidized CSE. These results demonstrated that the active vicinal thiols in the C252XXC255 motif can be oxidized to form disulfide by air oxygen.

Our previous study indicated that CSE activity can be regulated by a reversible redox change in the Cys252-X-X-Cys255 motif [20]. Luo et al. also reported that cysteine residue Cys252 in CSE could act as one of the predominant targets for persulfidation [22]. To verify whether cysteine residues in the Cys252-X-X-Cys255 motif are the target sites of persulfidation on CSE, the C252S and C255S variants were expressed in *E. coli*. The specific activities of the purified C252S and C255S mutants were 117.2 ± 16.66 nmol mg^−1^ min^−1^ and 123.3 ± 10.71 nmol mg^−1^ min^−1^, respectively, which were approximately 80% of the wild-type CSE activity (Figure S3). Next, we investigated the effect of persulfidation on the activity of CSE variants. The results showed that persulfidation inhibited the activities of the C252S and C255S mutant enzymes to a similar extent (approximately 40% residual activity), similar to the wild-type CSE. The activities of the persulfidated C252S and C255S mutants can also be reversed by the addition of DTT (Figure 1c,d).

**Figure 2:**
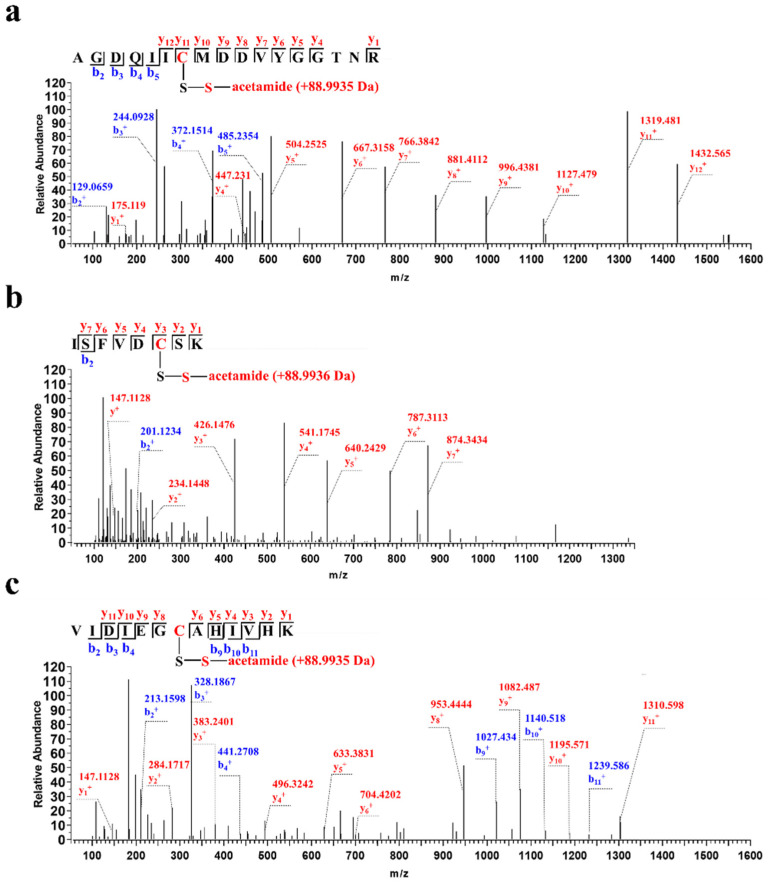
Identification of persulfidated cysteine(s) in CSE by mass spectrometry. (**a**) LC-MS/MS analysis of a trypsin-digested peptide containing the persulfidated Cys109 residue derivatized with iodoacetamide with an additional mass increment of 88.9935 Da. (**b**) A trypsin-digested peptide containing the persulfidated Cys137 residue (mass increment of 88.9936 Da) was identified. (**c**) A trypsin-digested peptide containing the persulfidated Cys172 residue (mass increment of 88.9935 Da) was identified.

Additionally, early reports indicated that H_2_S, more precisely the hydrosulfide anion HS- in equilibrium with it, can react with symmetric low-molecular-weight disulfides (RSSR) and mixed human serum albumin (HSA) disulfides [41,42]. Next, we determined whether H_2_S can reduce the disulfide bond in the Cys252-X-X-Cys255 motif involving the regulation of CSE activity. The activity of purified CSE (1 mg/mL) was not significantly affected by the treatment with 200 µM Na_2_S for 30 min at room temperature (Figure 1a). We also determined the number of free thiols in CSE treated with or without Na_2_S. A total of 7.8 ± 0.2 cysteines was modified per non-treated CSE monomer, similar to Na_2_S-treated CSE (7.9 ± 0.3 thiols/monomer), suggesting that the disulfide bond in the CXXC motif cannot be significantly reduced to form persulfides by H_2_S under these conditions. These data rule out the cysteines in the CXXC motif as sites for persulfidation modification and are consistent with the mass spectrometric data (Figure 2).

### 3.2. Identification of Persulfidated Cysteines

To identify which cysteine residues were persulfidated, purified wild-type CSE was treated with diamide and Na_2_S. Subsequently, the samples were alkylated with iodoacetamide followed by liquid chromatography–tandem mass spectrometry (LC-MS/MS) analysis. Three persulfidated cysteine residues, Cys109, Cys137 and Cys172, were identified. In the MS/MS spectrum, a 3^+^ charged ion (*m/z* 639.6027) corresponding to the trypsin-digested peptide AGDQIIC^109^MDDVYGGTNR (observed molecular mass, 1915.7847 Da; theoretical molecular mass, 1915.7863 Da) was identified (Figure 2a). The difference in the monoisotopic masses of the persulfidated peptide and the control underivatized peptide (triple-charged ion *m/z* 609.9382) is 88.9935 Da (3 × 29.6645 Da), which corresponds to the expected mass increment of the persulfidated Cys109 residue derivatized with iodoacetamide. Additionally, a 2^+^ charged ion (*m/z* 494.2173) corresponding to the peptide ISFVDC^137^SK (observed molecular mass, 986.4190 Da; theoretical molecular mass, 986.4201 Da) containing the persulfidated Cys137 residue with an additional mass increment of 88.9936 Da, (Figure 2b) and a 2^+^ charged ion (*m/z* 761.8789) corresponding to the peptide VIDIEGC^172^AHIVHK (observed molecular mass, 1521.7422 Da; theoretical molecular mass, 1521.7432 Da) containing the persulfidated Cys172 residue with an additional mass increment of 88.9935 Da were also identified (Figure 2c).

### 3.3. Cys137 Is Crucial for the Persulfidation of CSE

**Figure 3:**
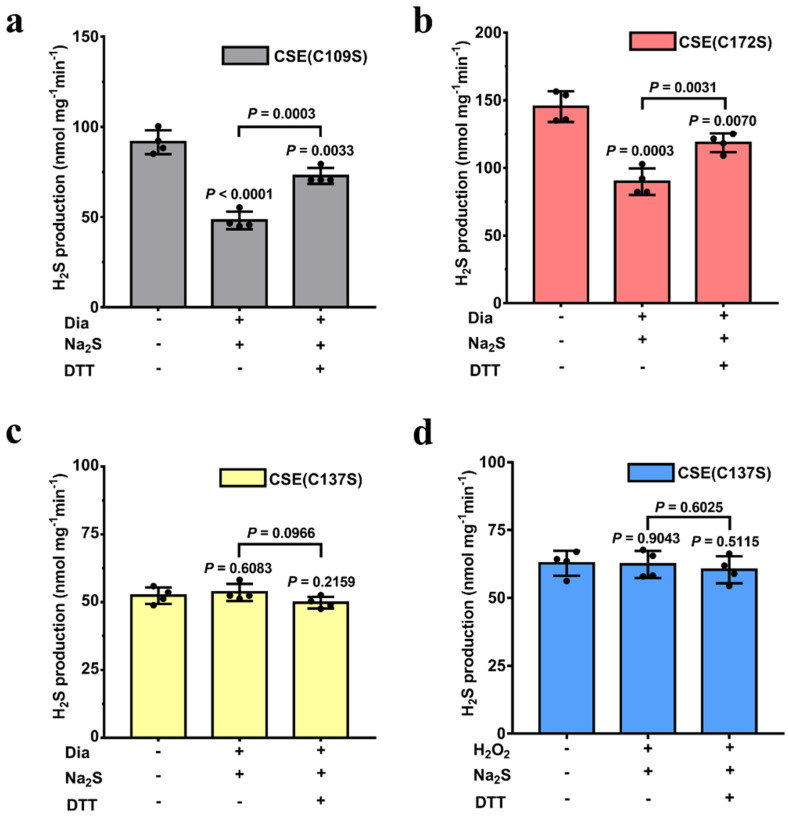
The Cys137 residue is crucial for the persulfidation of CSE. (**a**–**c**) Recombinant CSE variants CSE (C109S), CSE (C172S) and CSE (C137S) were incubated with diamide (Dia) and Na_2_S for persulfidation according to the legend of Figure 1. (**d**) Recombinant CSE (C137S) protein (1 mg/mL) was incubated with 50 µM H_2_O_2_ and 250 µM Na_2_S for persulfidation. DTT was used to reduce the persulfidated CSE. After the complete removal of DTT using a desalting column, the proteins were reoxidized by bubbling air through the solution. The data points and errors are the means ± SDs (n = 4).

According to mass spectrometric analysis, the C109S, C137S and C172S variants were expressed and purified (Figure S3). The H_2_S-producing activity of the C172S variant was 143.7 ± 6.48 nmol mg^−1^ min^−1^, similar to that of wild-type CSE. However, the remaining variants C109S and C137S were functionally impaired. The specific activities of the C109S and C137S variants were 88.7 ± 5.32 and 64.3 ± 7.20 nmol mg^−1^ min^−1^, respectively, and were approximately 58% and 42% of the wild-type CSE activity (Figure S3). These results are consistent with those reported by Fernandes et al. for the corresponding CSE variants [23]. Next, we assessed the effect of persulfidation on the activities of each CSE variant. As shown in Figure 3, the C109S and C172S variants exhibited a similar degree of inhibition as the wild-type CSE, with an activity value of 50–60% in persulfidation modification compared to the respective untreated CSE variant. Upon recovery by DTT, the enzyme activity was approximately 80% of the untreated enzyme activity. Conversely, no change in the activity of the C137S variant was observed before and after treatment by persulfidation. These results indicated that the Cys137 residue is crucial for the persulfidation of CSE.

**Figure 4:**
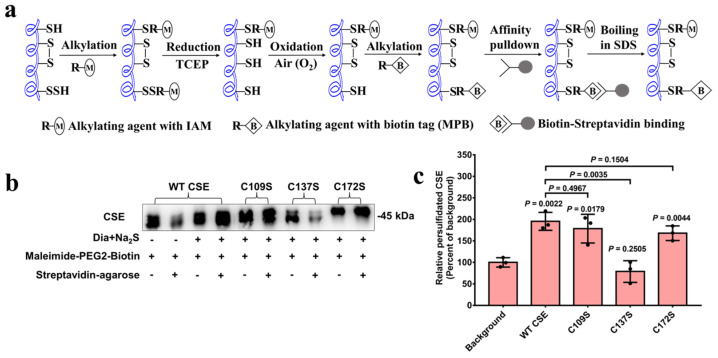
Identification of persulfidation levels of recombinant wild-type CSE and its variants. (**a**) protein persulfidation detection protocol. (**b**) Wild-type CSE (WT CSE) and its variants C109S, C137S and C172S were incubated with diamide and Na_2_S to generate persulfidated proteins, which were purified and detected by Western blot analysis with CSE antibody as described in the Materials and Methods section. Wild-type CSE untreated with diamide and Na_2_S was used as a background control. (**c**) Quantification of the persulfidated protein levels of wild-type CSE (WT CSE) and its variants, with data from (**b**) for comparison. ImageJ was used to analyze the intensities of Western blot bands for the persulfidated proteins. The data points and errors are the means ± SDs (n = 3) for three independent experiments.

To detect the persulfidation level of CSE, a convenient and reliable protocol based on streptavidin–biotin technology has been developed according to a previous report [39]. In the initial step, the cysteine thiol (-SH) and persulfide (-SSH) groups in CSE are alkylated using iodoacetamide (IAM). Subsequently, the derivatized persulfides and disulfides are reduced by the treatment with TCEP, but the thioethers (the thiol alkylation products) cannot be reduced. After the complete removal of the reductant, the active vicinal thiols Cys252 and Cys255 in CSE are reoxidized to form disulfide by bubbling air through the solution in the presence of trace amounts of metal ions [20,33,34,35]. The cysteine thiol groups that are generated from the derivatized persulfides are realkylated using the biotin-labeled alkylating agent EZ-Link Maleimide-PEG2-Biotin (MPB) and can thereafter be pulled down using streptavidin–agarose resin, followed by detection using SDS-PAGE upon their release from the resins by boiling in SDS containing a loading buffer (Figure 4a).

Other oxidized cysteine derivatives, such as cysteine sulfenic acids (Cys-SOH) and nitrosothiols (-SNO) may be alkylated to some extent, but these reactions would produce thioethers and hence cannot be reduced by TCEP. The persulfidated protein fractions of the wild-type CSE and the C109S, C137S and C172S variants were detected by Western blot analysis using a CSE antibody. As shown in Figure 4b,c, similar to the wild-type CSE, a similar degree of persulfidated protein was observed in the C109S and C172S variants, suggesting that Cys109 and Cys172 do not act as the primary sites for persulfidation modification. However, the protein persulfidation levels of the C137S variant markedly decreased compared to that of the wild-type CSE, indicating that the Cys137 residue is the crucial site for the persulfidation of CSE.

**Figure 5:**
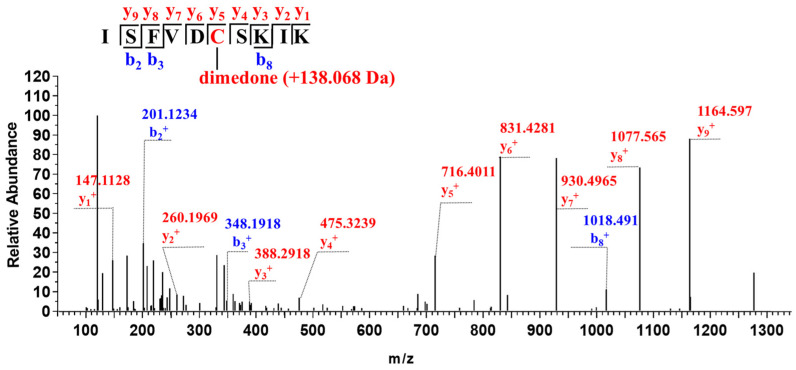
Identification of sulfenic acid modification of the residue Cys137 in CSE by mass spectrometry. LC-MS/MS analysis indicated the formation of a dimedone adduct (mass increment of 138.068 Da) of sulfenic acid in Cys137.

In addition to the reaction of H_2_S with diamide-induced mixed disulfides to form the persulfidated CSE, persulfidation modification can also be induced by H_2_S in reactive cysteine sulfenic acid (Cys-SOH), which is usually formed by the reaction of a thiol with hydrogen peroxide (H_2_O_2_) or other ROS [12]. In this study, the reactive cysteine thiol groups in recombinant CSE were oxidized to form the sulfenic acid (Cys-SOH) by H_2_O_2_ (50 µM). Subsequently, dimedone was added to the protein solutions to specifically label the cysteine sulfenic acid, followed by mass spectrometric analysis. As shown in Figure 5, only the Cys137 residue was identified as being modified with dimedone, thus indicating its sulfenic acid modification. The 2^+^ charged ion (*m/z* 639.3441) corresponding to the trypsin-digested peptide ISFVDC^137^SKIK (observed molecular mass, 1276.6726 Da; theoretical molecular mass, 1276.6737 Da) was identified. Cysteine sulfenic acid (Cys-SOH) modification at Cys137 was confirmed according to the dimedone mass increment (138.068 Da) observed at the y_5_^+^ ion (*m/z* 716.4011). Collectively, these results strongly indicated that the Cys137 residue in CSE is the primary site for persulfidation.

### 3.4. Increased Endogenous H_2_S Triggered by VEGF Promotes the Persulfidation Level of CSE in HA-VSMCs

**Figure 6:**
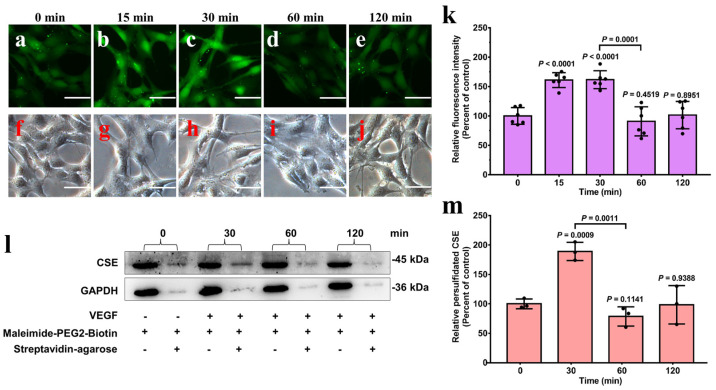
Increased endogenous H_2_S triggered by VEGF enhances the persulfidation level of CSE in HA-VSMCs. (**a**) Untreated cells imaged as described in the Materials and Methods section and were used as a control group. (**b**–**e**) Cells were incubated with VEGF (50 ng/mL) for 15, 30, 60 and 120 min at 37 °C and then imaged. (**f**–**j**) Bright field images corresponding to (**a**–**e**) (scale bar, 250 µm), respectively. (**k**) Quantification of the fluorescence intensities of H_2_S signaling with data from (**a**–**e**) for comparison. The graph represents the relative fluorescence intensity compared with that of the untreated cells (**a**) and shows the means ± SDs (n = 6). (**l**) Cells were incubated with VEGF (50 ng/mL) for 30, 60, and 120 min at 37 °C. The persulfidated proteins were purified using streptavidin–agarose resin, and the persulfidation levels of CSE were detected by Western blot analysis with CSE antibody as described in the Materials and Methods section. Cells not treated with VEGF were used as a control group. (**m**) Quantification of the persulfidated protein levels of CSE, with data from l for comparison. ImageJ was used to analyze the intensities of Western blot bands for the persulfidated CSE. The data points and errors are the means ± SDs (n = 3) for three independent experiments. GAPDH was used as a loading control.

Previous studies have indicated that VEGF treatment stimulates the endogenous H_2_S level via the enhancement of CSE activity in HA-VSMCs [36]. In our previous study, we also observed that cellular H_2_S production reached the highest level after VEGF treatment for 30 min and then gradually declined to the level of the untreated group, whereas the protein levels of CSE were not significantly changed [20]. We speculated that increased endogenous H_2_S in cells under oxidative stress conditions could inhibit the activity of CSE through persulfidation, thus leading to a decrease in cellular H_2_S levels. Therefore, we then investigated the effect of VEGF treatment on the levels of endogenous cellular H_2_S and persulfidated CSE. Expectedly, the endogenous H_2_S levels were significantly increased in cells that were incubated with 50 ng/mL VEGF for 15–30 min and then gradually decreased to the level of the control group (Figure 6a–k). Meanwhile, the persulfidated CSE in HA-VSMCs was detected at different time points (30–120 min) by Western blot analysis with CSE antibody. As shown in Figure 6l,m, HA-VSMCs exhibited a detectable basal level of persulfidation of CSE. The level of persulfidated CSE in VEGF-treated cells was consistent with the change tendency of endogenous cellular H_2_S levels. After VEGF treatment for 30 min, the persulfidation of CSE reached the highest level, followed by a return to baseline over time. Taken together, these experiments indicated that increased endogenous H_2_S in HA-VSMCs triggered by VEGF promotes the persulfidation of CSE.

## 4. Discussion

Growing evidence has indicated that H_2_S, as a signaling molecule, exerts a broad range of physiological effects, including the regulation of vascular tone, metabolism, apoptosis; the cellular stress response; and inflammation [1,2,6]. Maintaining the homeostasis of cellular H_2_S in different tissues plays an important role in the physiological functions of H_2_S. In previous studies, increased endogenous H_2_S generation by CSE was observed in HA-VSMCs incubated with VEGF for approximately 30 min. Subsequently, the H_2_S level gradually declined to the level of the control group after VEGF treatment for 1–2 h, whereas no significant difference was observed in the protein levels of CSE [20]. Given that the steady-state levels of H_2_S in cells have been shown to reflect a balance between its biosynthesis and its catabolism, we investigated whether VEGF treatment affects the protein level of ETHE1, which is proposed to oxidize the persulfide formed from H_2_S by SQR giving sulfite, thus resulting in the removal of H_2_S [5]. In fact, increased H_2_S levels were observed in ETHE1 knockout mice and patients with ETHE1 deficiency [43]. In the present study, VEGF treatment did not affect the protein level of ETHE1 (Figure S4), suggesting that the clearance rate of H_2_S may not be changed in HA-VSMCs. Based on these results and analysis, we speculated that increased endogenous H_2_S could feedback inhibit CSE activity via persulfidation, thus leading to a decrease in H_2_S levels and the maintenance of cellular H_2_S homeostasis. Previous studies have indicated that CSE is a target of persulfidation, while the key cysteine residues modified by H_2_S have not been systematically investigated, and the physiological functions remain poorly understood [22,24,44]. In the present study, we found that the H_2_S-producing activity of CSE is feedback-inhibited by its persulfidation at Cys137, and increased endogenous H_2_S promotes persulfidation of CSE in VEGF-treated cells.

Human CSE contains ten cysteine residues, including two highly conserved CXXC motifs, C252XXC255 and C307XXC310. A recent study from our lab revealed that H_2_O_2_-mediated oxidative stress enhances CSE-derived H_2_S synthesis through the oxidation of free thiols in the C252XXC255 motif to form the disulfide bond [20]. Additionally, previous studies have shown that the persulfidation of CSE probably occurs at cysteine residues in CXXC motifs [22,44]. To investigate whether the cysteines in the C252XXC255 motif are targets of persulfidation, we combined functional assays with mass spectrometric, mutagenesis and activity assays. Three cysteine residues at positions 109, 137 and 172 in CSE were identified as being modified with H_2_S using mass spectrometric analysis (Figure 2). However, only Cys137 was confirmed as a functional site for persulfidation using mutagenesis, activity assays (Figure 3) and streptavidin–biotin pull-down assays (Figure 4). These results rule out the cysteines in the C252XXC255 motif as functional sites for persulfidation. In fact, the reaction of H_2_S with typical disulfides is slow. The second-order rate constants for the reactions of H_2_S with typical low-molecular-weight (LMW) disulfides and the mixed albumin disulfides were in the order of 10^−2^ to 10^0^ M^−1^ S^−1^ at pH 7.4 [41,42].

Although some methods have been developed to detect the persulfides in biological systems, the quantification of intracellular protein persulfidation has remained problematic [12]. The modified biotin switch method has been questioned on chemical reaction mechanisms. In the tag-switch assay, the alkylating agent methylsulfonyl benzothiazole (MSBT) is not permeable to cell membranes. Furthermore, the selectivity of cleaving MSBT-labeled dialkyl disulfides over highly reactive protein disulfide moieties needs to be further assessed [39,45]. In another work by Nagy et al., an easy, convenient and highly specific method was used in determining persulfidated proteins, named ProPerDP. However, this method is only applicable for identifying the persulfidated proteins, which have only one surface-exposed thiol residue [39]. In the present study, we have developed a convenient and reliable protocol to detect and quantify persulfidated CSE (Figure 4a). The amino acid residue Cys137 in CSE has been identified as the predominant target for persulfidation modification (Figure 4b,c). The increased level of persulfidated CSE was observed in VEGF-treated cells. However, it should be noted that, in its current form, this method cannot distinguish between CSE persulfides and CSE polysulfide species. Although the polysulfidation of CSE was not identified by mass spectrometry in the purified recombinant CSE (Figure 2), it is not known whether or not CSE is polysulfidated under physiological conditions, and this issue needs to be investigated.

Recently, Fernandes et al. demonstrated that Cys137 is the main target residue of CSE nitrosation contributing to S-Nitrosoglutathione (GSNO)-mediated inhibition [23]. In the present study, the endogenous NO levels were not significantly affected in HA-VSMCs exposed to VEGF, suggesting that S-nitrosation modification should not be responsible for the activity regulation of CSE under current experimental conditions (Figure S5). Interestingly, the persulfidation and S-nitrosation of CSE occur at the same Cys137 residue and elicit similar effects on enzyme activity, suggesting the possibility that the H_2_S and NO signaling pathways compete for reactive cysteine residues in CSE. Based on the chemical reaction mechanism, it is not surprising that reactive cysteine residues in a set of targeted proteins act at the same site for persulfidation and S-nitrosation. For example, the persulfidation and S-nitrosation of GAPDH and nitric oxide synthase also occur in the same cysteine residues [24,46].

Persulfidation can be induced by H_2_S on reactive cysteine sulfenic acid (Cys-SOH) or by polysulfides on cysteine thiols [12]. Previous studies confirmed that VEGF stimulation of the VEGFR2 receptor can trigger an increased level of H_2_O_2_ derived from NADPH oxidases (NOX) [36,47,48,49], implying that the reactive cysteine thiol in CSE may be oxidized to cysteine sulfenic acid (Cys-SOH) under oxidative stress conditions. In fact, we observed that the ROS levels increased transiently before returning to steady-state levels in HA-VSMCs exposed to VEGF (Figure S6). Considering that the free thiol of Cys137 can be converted to sulfenic acid by treatment with H_2_O_2_, as evidenced by mass spectrometric analysis (Figure 5), it is predicted that the thiol of Cys137 should first be oxidized to sulfenic acid before being attacked by H_2_S. Actually, the pH-independent rate constant obtained for the H_2_S reaction with albumin sulfenic acid (RSOH) was in the order of 10^2^ M^−1^ S^−1^, which is about 2–4 orders of magnitude higher than that for H_2_S reactions with LMW disulfides and the mixed albumin disulfides [41,42]. In the cytosol, the reaction of H_2_S with sulfenic acids is kinetically challenged due to the concentration of oxidized thiols. Thus, the increased levels of oxidized thiols promote persulfides formation under oxidative conditions. Based on our experiments with cells treated with VEGF, the reaction of the increased levels of H_2_S with cysteine sulfenic acid (Cys-SOH) is a plausible pathway for persulfidation modification under oxidative stress conditions (Figure 6).

We sought to investigate the effect of the Cys137 residue on the structure and function of CSE. The crystal structure of human CSE (PDB: 2NMP) reveals that the Cys137 residue is located in a mobile loop region and is not particularly well exposed [9,23]. Since persulfidation is only detected at Cys137 and is functionally impaired upon its mutagenesis (Figure 3), protein dynamics could permit access to this reactive cysteine. In fact, although the amino acid residue Ser138 in CSE is not well exposed and located in the same loop as Cys137, O-GlcNAcylation of CSE at Ser138 was confirmed, and this modification promotes enzymatic activity to produce H_2_S [21]. Accordingly, the Cys137 residue should be oxidized by small molecule oxidants, such as endogenous H_2_O_2_, under physiological conditions.

In the crystal structure of human CSE, the cofactor PLP covalently attaches to the Lys212 residue, and the pyridine ring of PLP exhibits aromatic stacking interactions with the Tyr114 residue, which is located in the loop region Met110-Asn118 (Figure S7a,b). This loop forms a wall on the side of the PLP binding cleft in the active site, and its structural change has been predicted to affect enzyme activity [9]. The Cys137 residue is located in a mobile loop region very close to the loop Met110-Asn118 [23]. Therefore, it is plausible that persulfidation at the Cys137 position may structurally affect this loop region near the active site, thus leading to CSE inhibition. In addition, the distance between the Cys 137 residue and the C252XXC255 motif is ~30 Å, and it appears unlikely that persulfidation at the Cys137 residue regulates the CSE activity through a direct interaction with the C252XXC255 motif. These observations indicated that the H_2_S-producing activity of CSE is finely regulated by the C252XXC255 motif and persulfidation at Cys137, which stimulate and inhibit the activity, respectively. Interestingly, similar to the C252XXC255 motif in CSE, Cys137 is conserved in vertebrates, while it is absent in other eukaryotes (Figure S7c), suggesting that Cys137 might have specific biological functions. The detailed molecular mechanism and structural basis by which persulfidation at Cys137 inhibits CSE activity needs to be further investigated.

**Figure 7:**
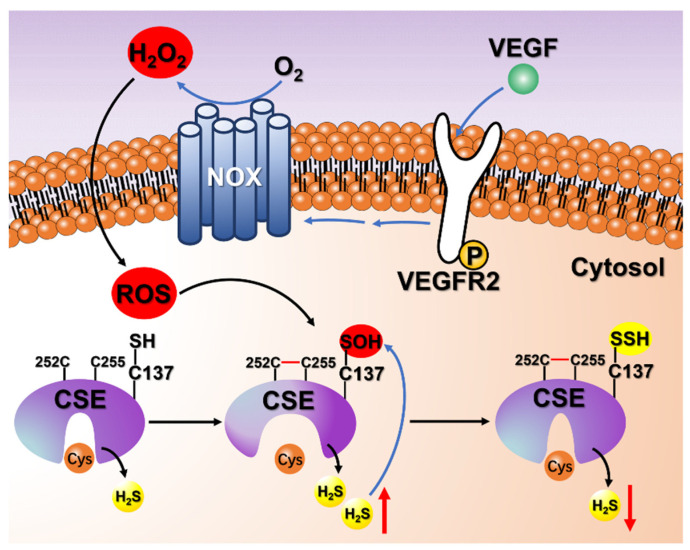
Schematic of the negative feedback regulation mechanism of the CSE/H_2_S system via persulfidation of CSE. VEGF stimulates the VEGFR2 receptor, which is autophosphorylated and then activates NOX to generate an increased level of endogenous H_2_O_2_. Increased cellular production of H_2_O_2_ or other ROS stimulates CSE activity by the oxidation of the free thiols to form a disulfide bond in the CXXC motif, thus leading to the enhancement of H_2_S production. Meanwhile, increased cellular production of H_2_O_2_ or other ROS also oxidizes Cys137 to form cysteine sulfenic acid (Cys-SOH), which reacts quickly with increased cellular H_2_S to form persulfidated CSE. Accordingly, persulfidation feedback inhibits the activity of CSE and results in a concomitant decrease in cellular H_2_S production. This regulatory mechanism may be partially responsible for maintaining cellular H_2_S homeostasis, particularly in tissues where CSE is a major source of H_2_S.

VEGF is a potent mitogen and angiogenic factor that has been shown to play a key role in neovascular responses that are involved in many physiological and pathological processes [50,51]. VEGF binds to tyrosine kinase cell receptors (VEGFRs), which are expressed predominantly in vascular endothelial cells, but can also be found in non-endothelial cells, such as vascular smooth muscle cells, inflammatory cells, and tumor cells [52,53,54]. Our previous study demonstrated that increased endogenous H_2_O_2_ triggered by VEGF can stimulate CSE activity in HA-VSMCs by the formation of a disulfide bond in the C252XXC255 motif, thus leading to an increase in H_2_S production [20]. Furthermore, increased endogenous H_2_O_2_ or other ROS could oxidize the free thiol of Cys137 in CSE to form cysteine sulfenic acid (Cys-SOH), followed by attack by increased cellular H_2_S or polysulfides. Consequently, persulfidation feedback inhibits CSE activity to concomitantly decrease CSE-derived H_2_S production (Figure 7). It is predicted that this synergetic regulation of CSE activity plays an important role in maintaining the steady-state level of H_2_S, particularly in tissues where CSE is a major source of H_2_S. Previous studies have shown that H_2_S is an endogenous stimulator of angiogenesis. The incubation of human umbilical vein endothelial cells (HUVECs) and human aorta smooth muscle cells with H_2_S activates ERK1/2 and p38 phosphorylation. These results implicate the regulation of H_2_S biosynthesis in the proangiogenic action of VEGF [55,56].

## 5. Conclusions

The maintenance of steady-state H_2_S concentrations is critical to its physiological functions. In addition to the clearance rate through the mitochondrial sulfide oxidation pathway, the activity of H_2_S-generating enzymes should be finely regulated. In this study, we report a negative feedback regulation mechanism of the CSE/H_2_S system via the persulfidation of CSE and demonstrate the potential for maintaining cellular H_2_S homeostasis under oxidative stress conditions, particularly in tissues where CSE is a major source of H_2_S.

## Data Availability

Data are contained within the article.

## Supplementary Materials

The following supporting information can be downloaded at https://www.mdpi.com/article/10.3390/antiox13111402/s1, Figure S1: Persulfidation of cystathionine γ-lyase (CSE) does not affect the content of PLP cofactor. Figure S2: Detection of the amount of inorganic polysulfides in Na_2_S solutions. Figure S3: Activities of the recombinant human wild-type CSE and its variants. Figure S4: Vascular endothelial growth factor (VEGF) treatment did not affect the protein level of persulfide dioxygenase (ETHE1). Figure S5: VEGF treatment does not affect the NO level of HA-VSMCs. Figure S6: VEGF treatment enhances the ROS level of HA-VSMCs. Figure S7: Structure and sequence alignment of human CSE.

## Abbreviations

CSE: cystathionine *γ*-lyase; CBS: cystathionine *β*-synthase; 3-MST: 3-mercaptopyruvate sulfur transferase; HA-VSMCs: human aortic vascular smooth muscle cells; GSNO: S-Nitrosoglutathione; GAPDH: glyceraldehyde-3-phosphate dehydrogenase; VEGF: vascular endothelial growth factor; PLP: pyridoxal 5′-phosphate; ETHE1: persulfide dioxygenase; LC-MS/MS: liquid chromatography–tandem mass spectrometry; CID: collision-induced dissociation; MPB: EZ-Link Maleimide-PEG2-Biotin; IAM: iodoacetamide; SQR: sulfide quinone reductase; DTNB: 5,5′-dithiobis(2-nitrobenzoic acid); TCEP: tris(2-carboxyethyl)phosphine.
