# Supplementary material for: Persulfidation of Human Cystathionine γ-Lyase Inhibits Its Activity: A Negative Feedback Regulation Mechanism for H2S Production
Source: Antioxidants (Basel). 2024 Nov 15;13(11):1402. doi: 10.3390/antiox13111402 (PMC11591423; doi:10.3390/antiox13111402)
Supplement: Supplementary file 1 [file antioxidants-13-01402-s001.zip › antioxidants-3270340-supplementary.pdf]

Persulfidation of human cystathionine γ-lyase inhibits its activity: A negative feedback regulation mechanism for H_2S production

Guanya Jia[#], Heng Li[#], Haisheng Gan, Jun Wang, Zhilong Zhu, Yanxiong Wang, Yongyi Ye, Xiaoya Shang, and Weining Niu*

School of Life Sciences, Northwestern Polytechnical University, Xi'an, 710072, China; jiagy@mail.nwpu.edu.cn (G.J.); aheng@mail.nwpu.edu.cn (H.L.); hsgan@mail.nwpu.edu.cn (H.G.); junw@mail.nwpu.edu.cn (J.W.); zhilongzhu_2019@mail.nwpu.edu.cn (Z.Z.); wangyanxiong2023@mail.nwpu.edu.cn (Y.W.); yyye@mail.nwpu.edu.cn (Y.Y.); loyamuyu@nwpu.edu.cn (X.S.)

* Correspondence: niuweining@nwpu.edu.cn; Tel.: +86-029- 88460386

# These authors contributed equally to this paper.

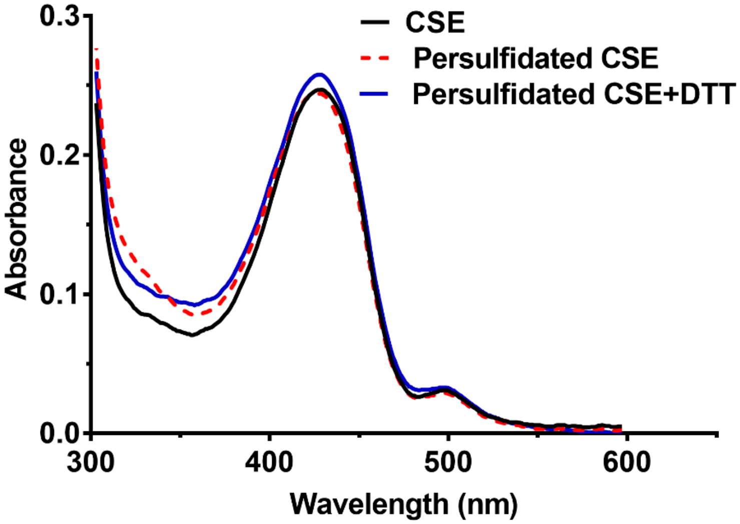

Figure S1. Persulfidation of cystathionine *γ*-lyase (CSE) does not affect the content of PLP cofactor. UV-visible absorption spectra of the purified CSE (solid black line), persulfidated CSE (dashed red line) and persulfidated CSE treated with 10 mM DTT (solid blue line). The protein concentration of each sample was adjusted to 1.5 mg/ml (50 mM Hepes buffer, pH 7.4).

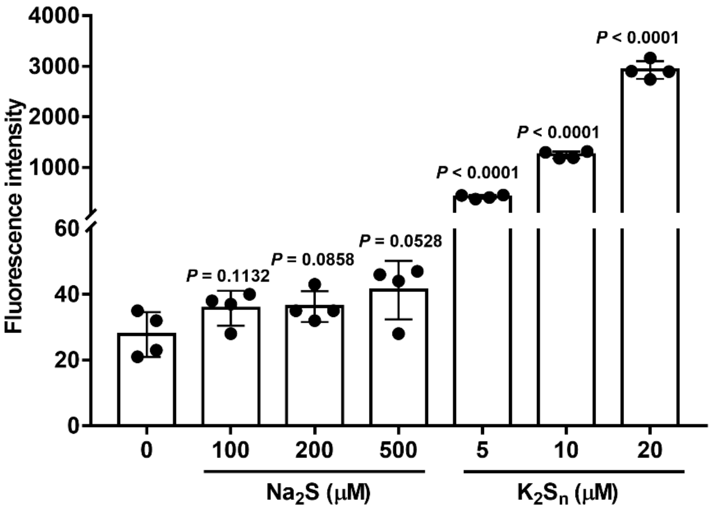

Figure S2. Detection the amount of inorganic polysulfides in Na_2S solutions. Reaction mixtures containing 50 mM HEPES buffer (pH 7.4), varying concentration of Na_2S or K_2S_n were incubated at room temperature for 30 min in a 96-well plate, and then, the fluorescent probe SSP4 was added to the mixtures at a final concentration of 10 μM. After incubation for 10 min at room temperature, the fluorescence intensities were measured at 515 nm (λ_{ex}=482 nm) by a multifunctional microplate reader. The data are presented as the means ± SD (n=4). An unpaired two-tailed Student's *t* test was employed to access the significance of differences between the fluorescence intensities of the control group (without Na_2S) and the experimental groups (with varying concentrations of Na_2S or K_2S_n).

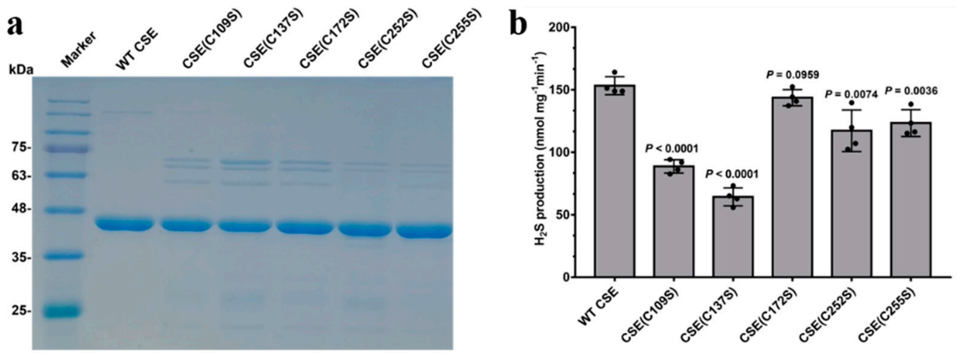

Figure S3. Activities of the recombinant human wild type CSE and its variants. **a**, The purified recombinant protein (5 μg) was analyzed by SDS-PAGE stained with Coomassie Blue. **b,** The H_2S-producing activities of wild type CSE(WT CSE), CSE(C109S), CSE(C137S), CSE(C172S), CSE(C252S) and CSE(C255S) were measured using 20 mM *L*-cysteine as substrates. The data are presented as the means ± SD (n=4). An unpaired two-tailed Student's *t* test was employed to access the significance of differences between the activities of CSE variant and wild type CSE.

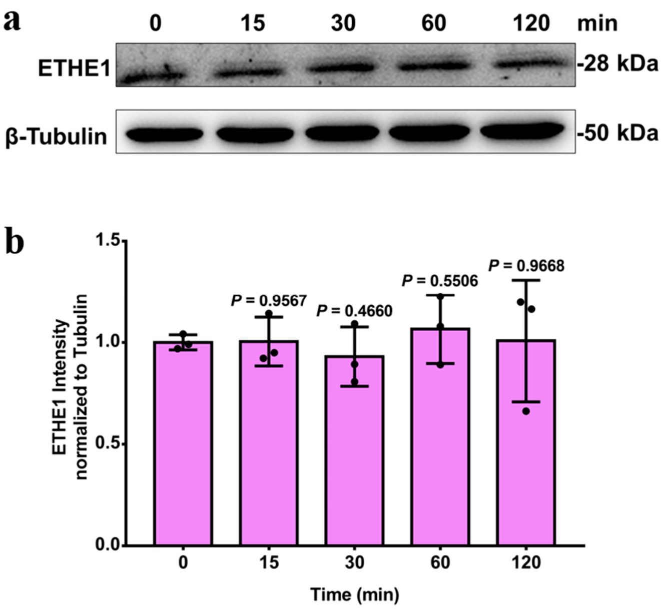

Figure S4. Vascular endothelial growth factor (VEGF) treatment did not affect the protein level of persulfide dioxygenase (ETHE1). a, Cells were incubated with VEGF (50 ng/ml) for 15, 30, 60 and 120 min at 37 ℃, respectively. The cell lysates (40 μg protein/lane) were resolved on SDS-PAGE and immunoblotted with ETHE1 antibody. **b**, Image J was used to analyze the intensities of western blot bands. The data points and errors are the means ± SD (n=3) for three independent experiments. β-tubulin was used as a loading control.

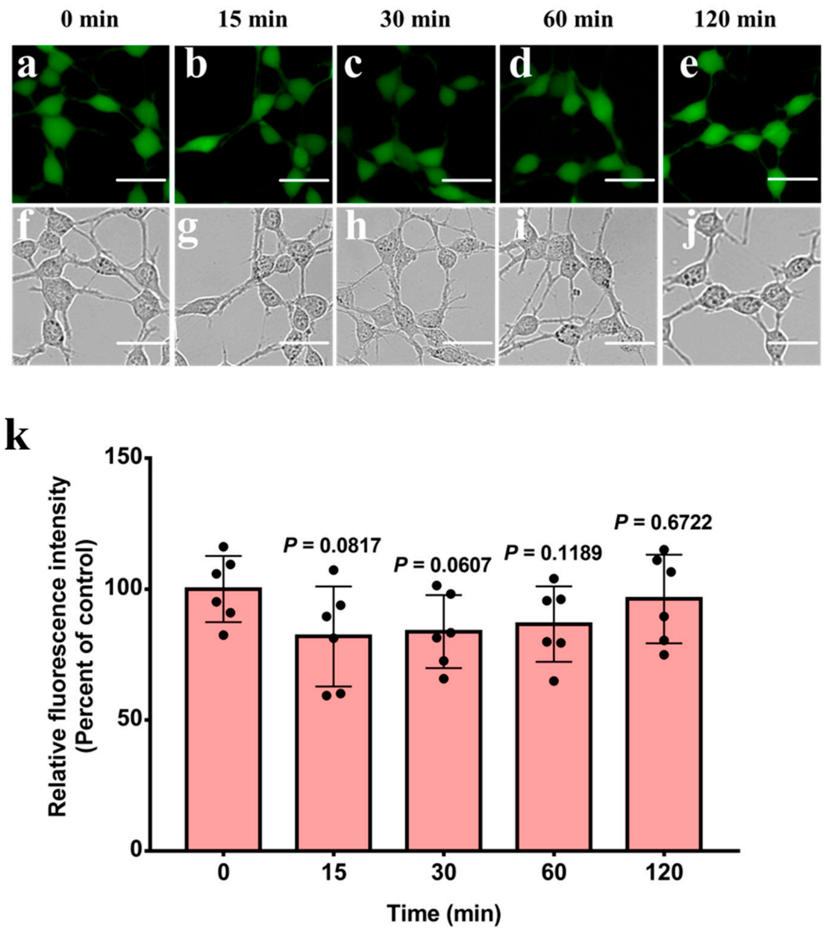

Figure S5. VEGF treatment does not affect the NO level of HA-VSMCs. a, Untreated cells imaged as described in the Materials and Methods section and were used as a control group. **b-e**, Cells were incubated with VEGF (50 ng/ml) for 15, 30, 60 and 120 min at 37°C and then imaged. **f-j**, Bright field images corresponding to a-e (scale bar, 250 μm), respectively. **k**, Quantification of the fluorescence intensities of NO signaling with data from a-e for comparison. The graph represents the relative fluorescence intensity compared with that of the untreated cells (a) and shows the means ± SDs (n=6).

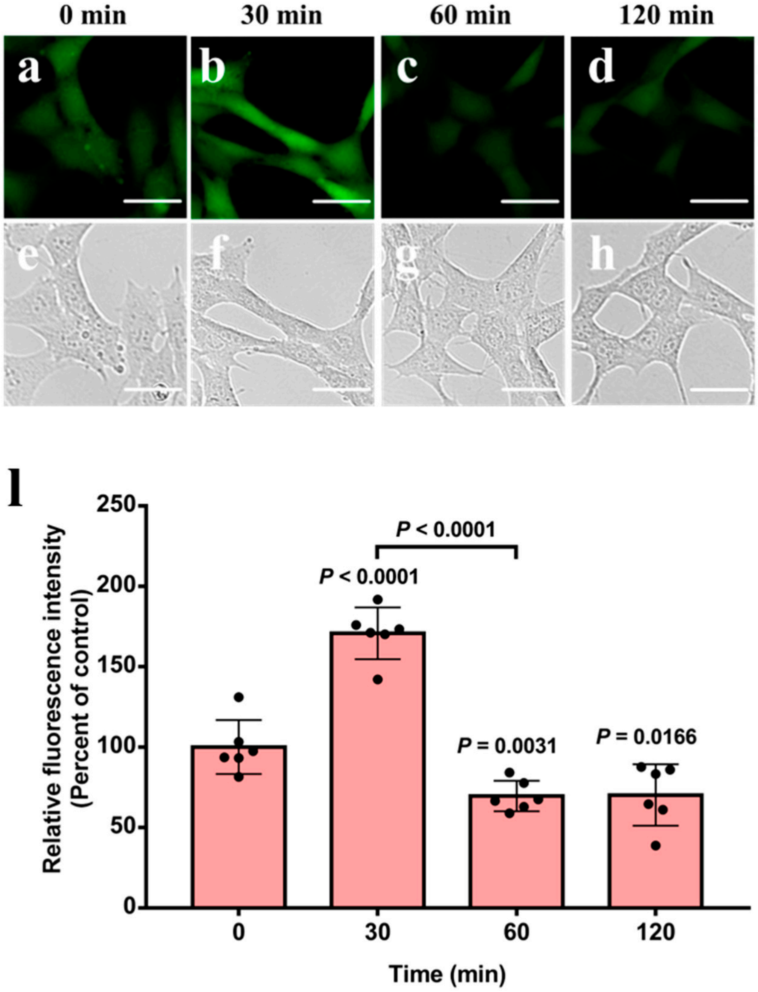

Figure S6. VEGF treatment enhances the ROS level of HA-VSMCs. a, Untreated cells imaged as described in the Materials and Methods section and were used as a control group. **b-d**, Cells were incubated with VEGF (50 ng/ml) for 30, 60 and 120 min at 37°C and then imaged. **e-h**, Bright field images corresponding to a-d (scale bar, 250 μm), respectively. **i**, Quantification of the fluorescence intensities of ROS signaling with data from a-d for comparison. The graph represents the relative fluorescence intensity compared with that of the untreated cells (a) and shows the means ± SDs (n=6).

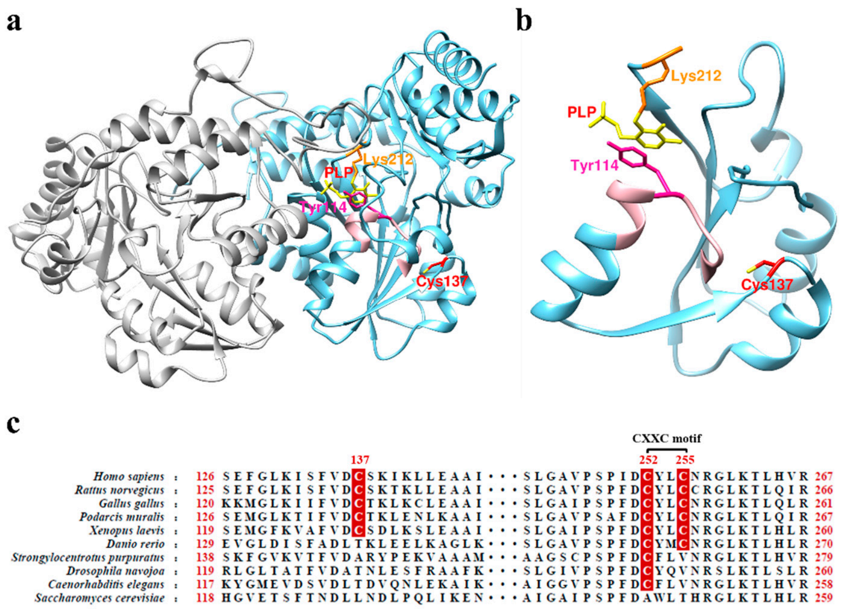

Figure S7. Structure and sequence alignment of human CSE. a, Cartoon representation of the crystal structure of human CSE (PDB ID: 2NMP). One subunit is colored in blue, while another subunit is shown in gray. **b**, Close view of the regions of the protein where the Cys137 is located. PLP cofactor (yellow sticks), Met110–Asn118 loop region (pink), Tyr114 residue (deep pink sticks), Lys212 residue (orange sticks) and Cys137 (red sticks) were shown. The figure was generated with UCSF Chimera. **c**, Alignment of amino acid sequences of CSE from different species using the Unipro UGENE software.
